# Caffeic Acid Counteracts LPS-Induced Inflammatory Damage in Yak Mammary Epithelial Cells Associated with NF-κB-Mediated Autophagy Regulation

**DOI:** 10.3390/ani16111605

**Published:** 2026-05-25

**Authors:** Yuan Li, Xupeng Li, Zhuo Chen, Ying Cen, Chunhai Zhang, Yufan Wang, Ruilan Zeng, Deyi Zhang, Xizhe Wang, Jian Li, Xianrong Xiong

**Affiliations:** 1 Key Laboratory for Animal Science of National Ethnic Affairs Commission, Southwest Minzu University, Chengdu 610041, China; yuanli102621@163.com (Y.L.); 18215163537@163.com (X.L.); zcheni4u@163.com (Z.C.); cy17208277814@163.com (Y.C.); zhangaotian007@163.com (C.Z.); yufanw0130@163.com (Y.W.); 18227689529@163.com (R.Z.); deyizhang@126.com (D.Z.); wangxizhe@163.com (X.W.); 2 Key Laboratory of Qinghai-Tibetan Plateau Animal Genetic Resource Reservation and Exploitation, Ministry of Education, Southwest Minzu University, Chengdu 610041, China; 3 Sichuan Zoige Alpine Wetland Ecosystem National Observation and Research Station, Southwest Minzu University, Chengdu 610041, China

**Keywords:** caffeic acid, LPS, yak mammary epithelial cells, inflammation, autophagy

## Abstract

Mastitis limits yak dairy production and is associated with lipopolysaccharide (LPS)-mediated inflammation in yak mammary epithelial cells (YMECs). This study aimed to investigate the protective effect of caffeic acid (CA) against LPS-induced cellular injury and to elucidate the underlying mechanisms, with a particular focus on autophagy regulation via the NF-κB signaling pathway. LPS exposure strikingly reduced cellular viability and increased intracellular reactive oxygen species (ROS) levels, accompanied by activation of the NF-κB pathway. Furthermore, it increased the expression of pro-inflammatory cytokines (TNF-α, IL-8, and IL-1β). In addition, LPS enhanced endoplasmic reticulum (ER) stress and Ca^2+^ dysregulation, increased LC3-II/LC3-I ratio, and reduced synthesis of α-casein and β-casein. Pretreatment with CA resulted in the effective alleviation of these alterations by restoring cellular viability, suppressing inflammatory responses, and normalizing autophagy-related markers. Additionally, inhibition of Nrf2 reversed the partial reversal of the protective effects of CA, resulting in increased ROS accumulation and autophagy activation, but did not impact NF-κB suppression. These findings indicate that CA attenuates LPS-induced inflammatory injury in YMECs involved in both Nrf2-dependent and independent pathways. These findings provide a mechanistic analysis of yak mastitis pathogenesis and CA potential as a natural therapeutic for improving mammary health and milk quality in yak dairy systems.

## Simple Summary

Mastitis is a disease affecting dairy herds, reducing milk yield and quality and causing economic losses, which seriously restricts the development of dairy. Mastitis is mainly caused by toxins of bacterial infections in yak populations that induce strong inflammatory responses in mammary cells. Current treatments mainly rely on antibiotics, but their long-term use raises concerns about drug resistance and food safety. Therefore, natural alternatives are of increasing interest in animal production. As a naturally occurring compound found in a variety of plant species, caffeic acid has been shown to possess a protective effect on mastitis. The findings of this study demonstrate that caffeic acid can reduce cell damage, decrease cellular stress levels, and restore the synthesis level of milk protein in cells. These findings suggest that caffeic acid has potential as a natural feed additive or therapeutic agent to improve udder health, enhance milk quality, and support more sustainable yak dairy production systems.

## 1. Introduction

The yak (*Bos grunniens*) has long been integral for the survival of local people. As a unique species adapted to the harsh environment of the Qinghai–Tibet Plateau. Yak milk and its derivatives serve as a primary source of nutrition for herdsmen due to their high-quality protein, earning them the moniker “nature’s concentrated milk” and income for local herders [1,2,3]. However, subclinical mastitis is relatively prevalent in yaks, accounting for approximately 36.6% [4]. The main pathogenic bacteria include *Escherichia coli* and *Staphylococcus aureus*. In extensively researched species such as cows and goats, it is known that mastitis impairs mammary epithelial cell function, reduces milk yield and quality, and inflicts substantial economic losses [5,6,7,8].

*Escherichia coli* is a major pathogen responsible for mastitis, and its endotoxin, lipopolysaccharide (LPS), plays a crucial role in initiating immune responses. LPS, a potent inducer of inflammation, binds to Toll-like receptor 4 (TLR4) on the surface of both immune and epithelial cells or stimulates the generation of reactive oxygen species (ROS), activating the nuclear factor-kappa B (NF-κB) pathway, thereby stimulating the expression of pro-inflammatory cytokines, including TNF-α, IL-1β, and IL-6 [9,10,11,12,13]. Furthermore, LPS-induced signaling is closely associated with intracellular stress responses, such as endoplasmic reticulum (ER) stress, calcium dysregulation, and autophagy imbalance [14,15,16]. These changes promote epithelial injury, ultimately resulting in tissue dysfunction and exacerbating the severity of mastitis.

At the cellular level, inflammatory stimulation and environmental stress factors often induce endoplasmic reticulum stress and activate autophagy. These stress responses are closely linked to autophagy. These two mechanisms are intricately linked and play a pivotal role in preserving cellular homeostasis [17,18,19,20]. Autophagy, for instance, mainly works to relieve oxidative stress by clearing damaged organelles and dialing down inflammation. In fact, it has been shown to keep the NF-κB and NLRP3 pathways in check, which is key for maintaining a stable intracellular environment [21]. The relationship between ER stress and autophagy is bidirectional. ER stress stimulates autophagy through the action of transcription factors such as CHOP and ATF4. Instead, autophagy mitigates ER stress by clearing misfolded proteins and damaged organelles [22,23]. However, the contribution of autophagy imbalance to yak mastitis remains unclear.

Nuclear factor erythroid 2-related factor 2 (Nrf2) regulates cellular antioxidant defense. Nrf2 coordinates the antioxidant response by enhances the expression of enzymes such as heme oxygenase-1 (HO-1) and nicotinamide quinone oxidoreductase-1 (NQO1) to protect cells from oxidative damage. Beyond that, Nrf2 also helps orchestrate autophagy by modulating key genes such as LC3 and p62. thereby regulating oxidative stress, ER stress, and autophagy in a coordinated response to cellular damage [24,25,26,27]. Nevertheless, the role of Nrf2 in LPS-induced autophagy imbalance in yak mammary epithelial cells (YMECs) has not been defined.

In recent years, there has been an increasing interest in the potential of natural phenolic compounds to mitigate LPS-induced cellular damage [15,16]. Caffeic acid (CA), a widely distributed phenolic acid found in various herbs, exhibits strong antioxidant and anti-inflammatory properties [28]. Previous studies show that CA regulates oxidative stress and inflammatory reactions by reducing the expression of pro-inflammatory factors, such as IL-8 and IL-1β, and inhibiting the expression of NF-κB, thereby reducing the damage to mammary epithelial cells induced by LPS [29,30,31]. These findings suggest that CA has therapeutic potential in combating the detrimental effects of LPS in mammary cells. However, current studies have mainly focused on mouse or bovine models, while the effects and underlying mechanisms of CA in yak mammary epithelial cells remain poorly understood. Whether caffeic acid protects yak mammary epithelial cells by regulating NF-κB–associated autophagy imbalance remains unknown.

Therefore, this study investigated the protective effects of caffeic acid against LPS-induced inflammatory injury in YMECs, with a particular focus on the interplay between NF-κB signaling, autophagy, and Nrf2 pathways. These findings may provide new insights into the pathogenesis of yak mastitis and identify CA as a potential natural therapeutic agent for improving mammary health in yak dairy systems.

## 2. Materials and Methods

### 2.1. Cell Culture

Primary yak mammary epithelial cells (YMECs) were isolated from healthy lactating adult female yaks without mastitis, characterized by mammary glands with no evident redness or swelling and a soft tissue texture, using an enzymatic digestion method. The culture procedure was performed according to the previously established protocol by Fu et al. [32] with minor modifications. After mincing, the pieces of tissue were digested with 0.1% type IV collagenase (C8161, Solarbio, Beijing, China), at 37 °C for 30 min in a water bath. Cells were subjected to wash twice with 2 mL of 2X PBS and pelleted upon centrifugation at 3000× *g* rpm for 5 min. The DMEM/F12 medium was supplemented with 10% fetal bovine serum (FBS), 0.5% insulin-transferrin-selenium (ITS), 10 ng/mL epidermal growth factor (EGF), 2.5 μg/mL amphotericin, 2% penicillin-streptomycin, and 1 μg/mL hydrocortisone. Cells were incubated at 37 °C in a 5% CO_2_ atmosphere. The isolated YMECs were cryopreserved in liquid nitrogen at −196 °C. Prior to experiments, the cells were thawed and seeded in culture medium. To induce an inflammatory response, cells at passage 3 were treated with lipopolysaccharide (LPS, 0.1, 1, 5, 10 μg/mL) to identify the optimal concentration for model establishment, and caffeic acid was applied at varying concentrations (75, 150, 300 μM) to evaluate its protective effects, cells were pretreated with CA for 6 h, followed by co-treatment with LPS for 48 h.

### 2.2. Enzyme-Linked Immunosorbent Assay (ELISA)

Culture supernatants were collected from different treatment groups (*n* = 3 each group) and analyzed for concentrations of IL-1β (MM-3694901), IL-8 (MM-3695101), IL-10 (MM-3695201), TNFα (MM-156801), α-casein (MM-5101701), β-casein (MM-5047101), and κ-casein (MM-5047501) using Bovin Autoimmune Response ELISA Kits (Meimian, Yancheng, China). All ELISA procedures were performed according to the manufacturer’s instructions. Absorbance was measured at 450 nm using a microplate reader (SpectraMaxSky, Thermo Scientific, Waltham, MA, USA). Each sample was assayed in triplicate.

### 2.3. Fluorescence Staining

Following cellular treatments, the cells were washed with PBS and then incubated with appropriately diluted fluorescent probes under light-protected conditions at the recommended temperature. Intracellular ROS levels were detected using a ROS assay kit (CA1420, Solarbio, Beijing, China), and calcium levels were measured with a Ca^2+^ assay kit (S1061, Beyotime, Shanghai, China) in accordance with the manufacturers’ protocols. For endoplasmic reticulum visualization, cells were stained with ER-Tracker Red (C1041S, Beyotime, Shanghai, China) and observed under a fluorescence microscope (Observer Z1, Zeiss, Oberkochen, Germany). Fluorescence intensity was quantified using ImageJ 1.52a software (National Institutes of Health, Bethesda, MD, USA).

### 2.4. TUNEL Assay

Apoptosis was evaluated using the terminal deoxynucleotidyl transferase dUTP nick-end labeling (TUNEL) assay with a TUNEL FITC Apoptosis Detection kit (A111, Vazyme, Nanjing, China) according to the manufacturer’s instructions. Briefly, cells were fixed with 4% paraformaldehyde, permeabilized with 0.2% Triton X-100, and subsequently incubated with the TdT reaction mixture at 37 °C for 60 min in a humidified, light-protected chamber. After extensive washing, nuclei were counterstained with DAPI. Samples were examined by fluorescence microscopy, and apoptotic nuclei were identified based on FITC-positive signals.

### 2.5. Quantitative Real-Time PCR Analysis (qRT-PCR)

Total RNA was isolated from YMECs using Trizol reagent (Invitrogen, Carlsbad, CA, USA) according to the manufacturer’s instructions. RNA purity and concentration were assessed with a NanoDrop spectrophotometer (Thermo Scientific, Waltham, MA, USA). Complementary DNA (cDNA) was synthesized with the Transcriptor First Strand cDNA Synthesis Kit (R312-01/02, Vazyme, Nanjing, China) as the manufacturer’s guidelines. Quantitative real-time PCR (qRT-PCR) was conducted on a LightCycler system (Roche, Basel, Switzerland) following the manufacturer’s protocol. The primer sequences used are listed in Table 1, and their specificity was verified by melting curve analysis. All primers were designed in-house as described in Table 1. β-actin was used as an internal control to normalize of target mRNAs expression levels. The relative expression of target mRNAs was calculated using the 2^−ΔΔCt^ method, with all experiments performed in triplicate.

### 2.6. Western Blotting Analysis

Protein was extracted from cells using a protein extraction kit (P0013M, Beyotime, Shanghai, China). Protein concentration was determined with a BCA kit (P0012S, Beyotime, Shanghai, China). A total of 30 μg of protein per sample was separated by 10% SDS-PAGE and transferred onto PVDF membranes. The membranes were then blocked with 5% BSA and incubated with primary antibodies targeting the NF-κB (1:500, ER0815, HUABIO, Hangzhou, China), p-NF-κB (1:500; HA723223, HUABIO, Hangzhou, China), Nrf2 (1:1000; HA721432, HUABIO, Hangzhou, China), p62 (1:1000, A22025, Abclonal, Wuhan, China), LC3 (1:1000, A19665, Abclonal, Wuhan, China), and Beclin-1 (1:200, A17028, Abclonal, Wuhan, China). β-actin was used as an internal control to normalize of target protein expression levels. Subsequently, the membranes were incubated with corresponding secondary antibodies, and protein signals were detected using an ECL chemiluminescence reagent.

### 2.7. Immunofluorescence Staining

The collected mammary gland cells were fixed with 4% paraformaldehyde, then permeabilized with an appropriate amount of Triton X-100 (Beyotime, P0096, Shanghai, China) for 20 min at room temperature. Nonspecific binding sites were blocked with QuickBlock^TM^ (Beyotime, P0260, Shanghai, China) for 30 min. The cells were then incubated overnight at 4 °C with different primary antibodies. The primary antibodies used in this study were as follows: Phospho-NF-kB p65 (1:200), anti-NF-κB p65 (1:200), and LC3 (1:200). After five washes with PBS, the cells were incubated with a secondary antibody (1:800, ab150080, Abcam, Waltham, MA, USA) at for 2 h at room temperature in the dark. Following PBS washes, nuclei were stained with DAPI for 5 min while protected from light. Finally, the samples were observed as soon as possible.

### 2.8. Statistical Analysis

Data are presented as the mean ± standard error of the mean (SEM). Statistical analyses were performed using GraphPad Prism 10 (GraphPad Software, San Diego, CA, USA). For comparisons involving more than two groups, one-way or two-way ANOVA was employed, followed by Dunnett’s post hoc test as appropriate for the experimental design. Statistical significance is indicated as follows: * *p* < 0.05, ** *p* < 0.01, and *** *p* < 0.001.

## 3. Results

### 3.1. LPS Induces Inflammatory Injury in YMECs

**Figure 1:**
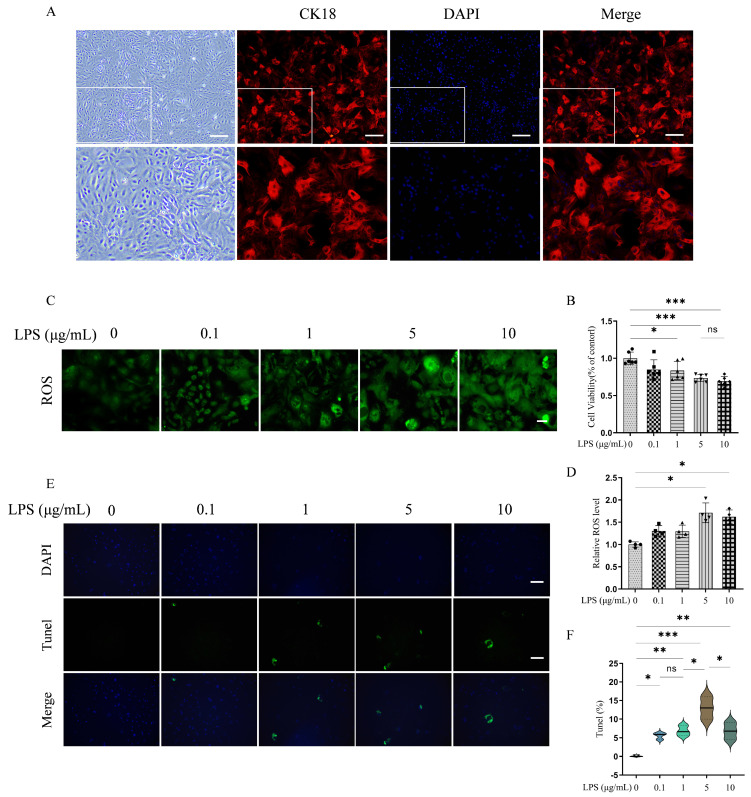
Establishment of the LPS-induced inflammatory injury model of YMEC. Cells were pretreated with different concentrations of LPS (0.1, 1, 5, and 10 μg/mL) for 48 h. (**A**) Identification of CK18 in YMEC by immunofluorescence staining. Nuclei, blue; CK18, red; Scale bar = 10 μm. (**B**) YMECs were treated with LPS for 48 h, and the cytotoxicity of LPS was detected using the CCK-8 assay (*n* = 6). (**C**) ROS levels were measured by DCFH-DA, Scale bar = 10 μm. (**D**) Quantification of ROS levels (*n* = 4). (**E**) Representative images presenting cell apoptosis by the Tunel assay. (**F**) Statistics of apoptosis rate. One-way ANOVA, Dunnett’s post hoc test. Data are presented as the mean ± SEM. ns *p* > 0.05 (not significant), * *p* < 0.05, ** *p* < 0.01, *** *p* < 0.001.

**Figure 2:**
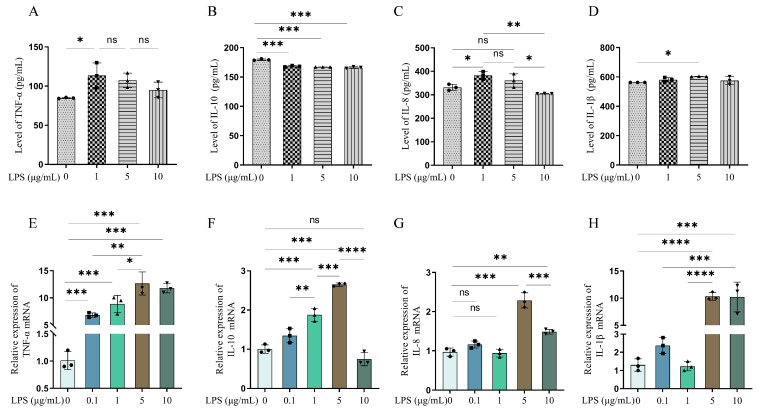
Establishment of the LPS-induced inflammatory injury model of YMEC. Cells were pretreated with different concentrations of LPS (0.1, 1, 5, and 10 μg/mL) for 48 h. (**A**–**D**) TNF-α, IL-1β, IL-10, and IL-8 enzymatic activity content of immune factors (*n* = 3). (**E**–**H**) qRT-PCR analysis of relative mRNA levels of inflammatory factors (*n* = 3). One-way ANOVA, Dunnett’s post hoc test. Data are presented as the mean ± SEM. ns *p* > 0.05 (not significant), * *p* < 0.05, ** *p* < 0.01, *** *p* < 0.001, **** *p* < 0.0001.

To confirm the epithelial identity of the cultured cells, CK18 immunoreactivity was verified by immunofluorescence staining (Figure 1A). We next evaluated the effects of LPS on cellular injury in YMECs by assessing cell viability, ROS accumulation, as well as the activity and transcriptional expression of signature cytokines. After 48 h of exposure to LPS at concentrations of 0.1, 1, 5, and 10 µg/mL, a progressive decline in cell viability was observed with increasing concentration. Significant decreases in cell viability were observed at 5 and 10 µg/mL LPS (Figure 1B; *p* < 0.001). LPS exposure also increased intracellular oxidative stress. Treatment with 5 µg/mL and 10 µg/mL LPS sharply elevated ROS levels in YMECs (Figure 1C,D; *p* < 0.001). TUNEL staining further demonstrated that 5 μg/mL LPS significantly increased apoptosis in YMECs (Figure 1E,F; *p* < 0.01). We further examined inflammatory cytokine production in response to LPS. Compared with the 0 μg/mL control group, treatment with 1 μg/mL of LPS resulted in increased enzymatic activity of TNF-α and IL-8 (Figure 2A,C; *p* < 0.05), whereas treatment with 5 μg/mL of LPS selectively increased IL-1β levels (Figure 2D; *p* < 0.05). In contrast, the anti-inflammatory cytokine IL-10 was consistently reduced across all concentrations (Figure 2B; *p* < 0.001). At the transcriptional level, LPS treatment robustly elevated TNF-α and IL-10 mRNA levels at all concentration (Figure 2E,G; *p* < 0.001). 5 μg/mL and 10 μg/mL LPS further induced IL-8 and IL-1β transcripts (Figure 2F,H; *p* < 0.001). Although both 5 and 10 µg/mL LPS elicited robust inflammatory responses, the 5 µg/mL dose yielded the strongest mediator induction and was therefore selected for subsequent model establishment.

### 3.2. Caffeic Acid Protects YMECs from LPS-Induced Inflammatory Responses

**Figure 3:**
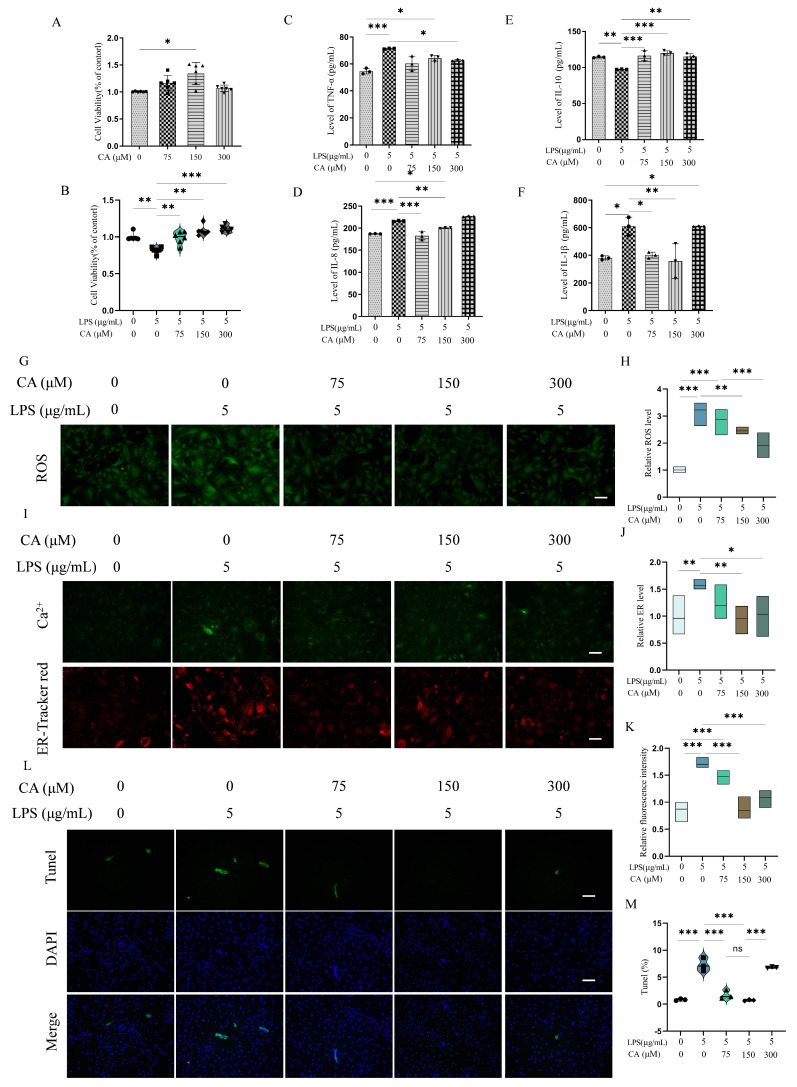
CA alleviates LPS-induced inflammatory response. Cells were pretreated with different concentrations of CA (75, 150, and 300 μM) for 54 h in the absence or presence of LPS (5 μg/mL) for 48 h. (**A**) YMEC was treated with CA for 54 h, and the cell vitality was detected using CCK-8 assay (*n* = 6). (**B**) Cells were pretreated with different concentrations of CA (75, 150, and 300 μM) for 54 h in the absence or presence of LPS (5 μg/mL) for 48 h, and the Cell vitality was detected using CCK-8 assay (*n* = 6). (**C**–**F**) TNF-α, IL-1β, IL-10, and IL-8 enzymatic activity content of immune factors (*n* = 3). (**G**) ROS levels were measured by DCFH-DA, Scale bar = 100 μm. (**H**) Quantification of ROS levels. (**I**) Representative images presenting ER and Ca^2+^ staining images. (**J**,**K**) Quantification of ER and Ca^2+^ concentration levels. (**L**) Representative images presenting cell apoptosis by the Tunel assay, Scale bar = 10 μm. (**M**) Statistics of apoptosis rate. One-way ANOVA, Dunnett’s post hoc test. Data are presented as the mean ± SEM. ns *p* > 0.05 (not significant), * *p* < 0.05, ** *p* < 0.01, *** *p* < 0.001.

We next examined the protective effects of caffeic acid on LPS-induced injury in YMECs by evaluating changes in cell viability, cytokine secretion, oxidative stress markers, ER stress, Ca^2+^ homeostasis, and apoptosis were analyzed as experimental design. In the present study, CA was co-cultured with YMECs for 54 h. As shown in Figure 3A, treatment with 150 μM CA resulted in a significant enhancement of YMECs viability in comparison with the 0 μM CA group (*p* < 0.05).

Following the addition of LPS after a 6 h pretreatment with CA, a significant alleviation of the LPS-induced decline in cell viability was exhibited by CA at various concentrations, in comparison to the LPS group (Figure 3B; *p* < 0.01). In contrast to the CON group, the LPS-treated group demonstrated heightened levels of IL-1β, TNF-α, and IL-8 expression accompanied by suppressed IL-10 expression (*p* < 0.01). CA pretreatment at concentrations of 75 and 150 μM selectively down-regulated levels of IL-8 and IL-1β without affecting TNF-α levels. In contrast, the 300 μM group significantly decreased TNF-α levels leaving IL-8 and IL-1β unchanged. Remarkably, all doses CA pre-treatment restored IL-10 secretion (Figure 3C–F; *p* < 0.01). In parallel, LPS exposure resulted in simultaneously elevated intracellular ROS levels (Figure 3G,H; *p* < 0.01), augmented endoplasmic reticulum (ER) stress, and provoked Ca^2+^ efflux (Figure 3I–K; *p* < 0.01). CA Pretreatment at concentrations of 150 and 300 μM significantly reduced ROS accumulation, alleviated ER stress, and limited Ca^2+^ leakage, compared with the LPS group. We next evaluated apoptotic responses. As shown in Figure 3L,M, TUNEL analysis showed that CA significantly reduced LPS-induced apoptosis (*p* < 0.001), with no significant difference observed between 75 and 150 μM (*p* > 0.05). Collectively, 150 μM CA emerges as the minimal fully effective dose and was adopted for mechanistic studies of its mechanism of action.

### 3.3. Caffeic Acid Suppresses NF-κB Activation in LPS-Induced YMECs

**Figure 4:**
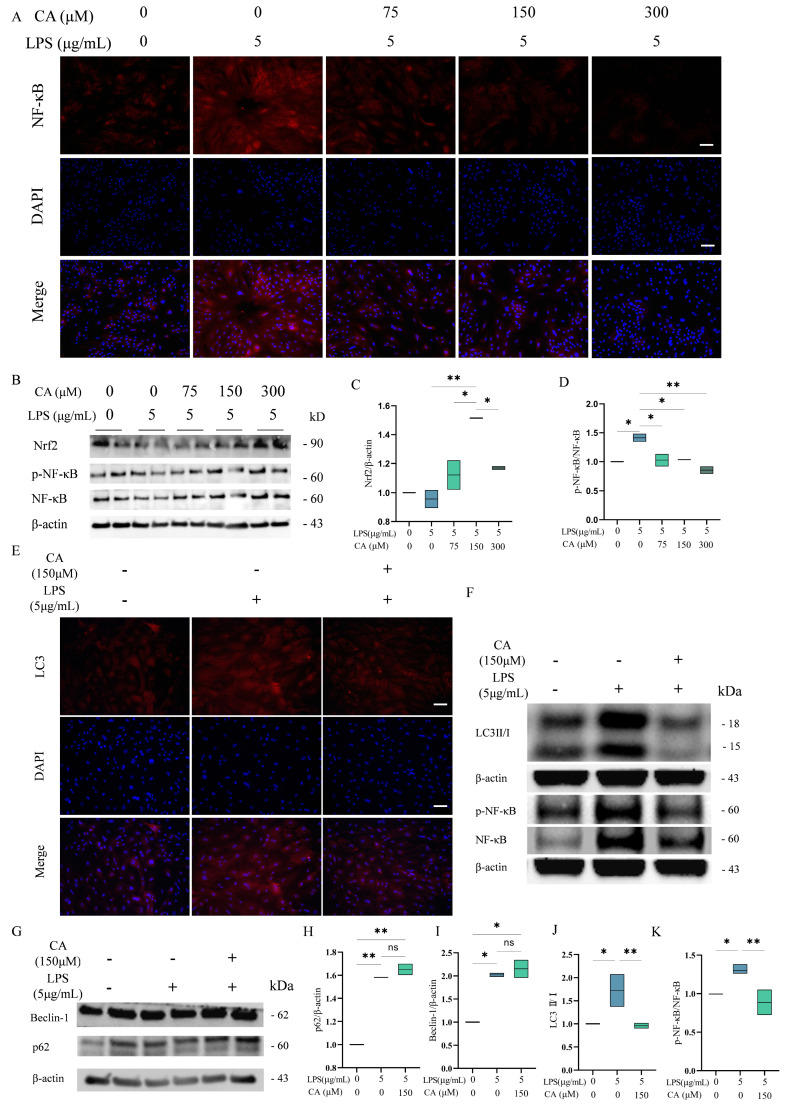
CA alleviates LPS-induced inflammatory response. Cells were pretreated with different concentrations of CA (75, 150, and 300 μM) for 54 h in the absence or presence of LPS (5 μg/mL) for 48 h. (**A**) Immunofluorescence result for NF-κB, Scale bar = 10 μm. (**B**–**D**) Western blot analysis of Nrf2, NF-κB, and pNF-κB protein. (**E**) Immunofluorescence results for LC3, Scale bar = 10 μm. (**F**,**J**,**K**) Western blot analysis of LC3, NF-κB, and p-NF-κB protein. (**G**–**I**) Western blot analysis of Beclin-1, p62 protein. One-way ANOVA, Dunnett’s post hoc test. Data are presented as the mean ± SEM. ns *p* > 0.05 (not significant), * *p* < 0.05, ** *p* < 0.01.

To further validate the cytoprotective role of CA, subsequent experiments focused on its regulatory effects on key inflammatory and autophagy-related signaling pathways. As demonstrated in Figure 4, the LPS-injured group displayed a marked upregulation of NF-κB protein compared with the control group. Pretreatment with CA at all tested concentrations significantly attenuated NF-κB activation induced by LPS (*p* < 0.05), no significant differences were observed among the CA-treated groups. Conversely, LPS exposure showed a tendency to decrease Nrf2 protein expression, although this change did not reach statistical significance. Notably, pretreatment with 150 μM CA (CA150 group) significantly increased Nrf2 protein expression compared to the LPS-treated group (*p* < 0.05). Based on these findings, 150 μM CA was selected as the optimal concentration for subsequent mechanistic investigations.

### 3.4. Caffeic Acid Suppresses Excessive Autophagy and Preserves Epithelial Function in LPS-Stimulated YMECs

**Figure 5:**
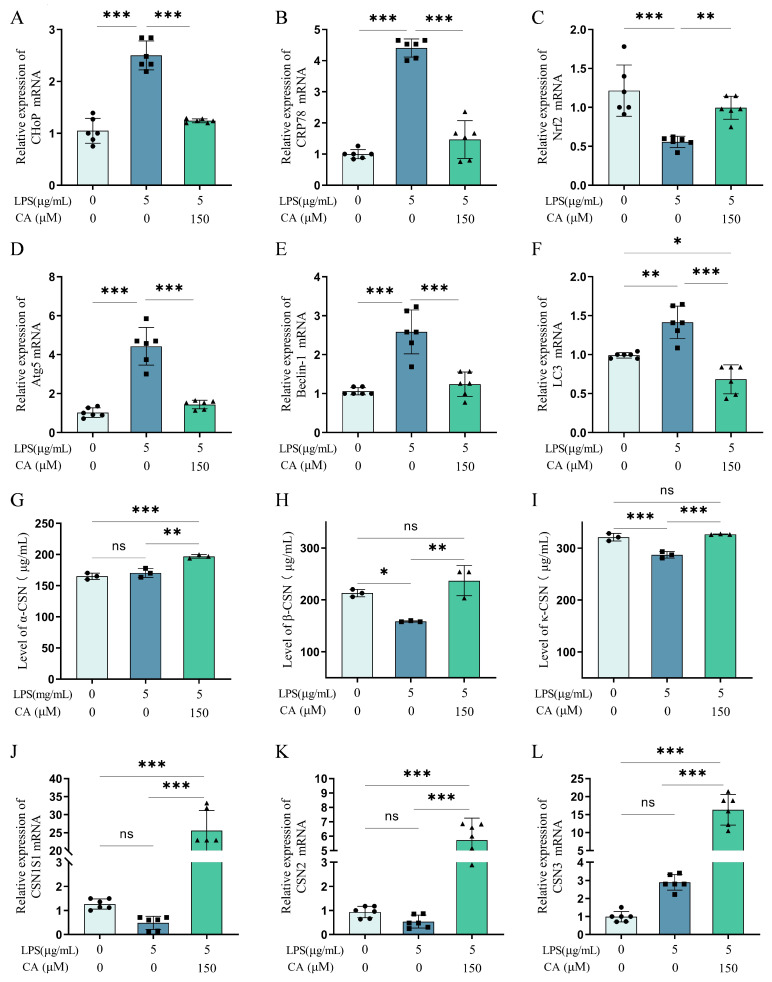
CA alleviates the inflammatory responses induced by LPS, reduces endoplasmic reticulum stress and autophagy, and affects casein content. Cells were pretreated with CA (150 μM) for 54 h in the absence or presence of LPS (5 μg/mL) for 48 h. (**A**,**B**,**D**) qRT-PCR analysis of relative mRNA levels of endoplasmic reticulum (*n* = 6). (**C**) qRT-PCR analysis of relative mRNA levels of *Nrf2* (*n* = 6). (**E**,**F**) qRT-PCR analysis of relative mRNA levels of autophagy (*n* = 6). (**G**–**I**) Quantification of *α-CSN*, *β-CSN*, and *κ-CSN* content (*n* = 3). (**J**–**L**) qRT−PCR analysis of relative mRNA levels of *α-CSN*, *β-CSN*, and *κ-CSN* (*n* = 6). One-way ANOVA, Dunnett’s post hoc test. Data are presented as the mean ± SEM. ns *p* > 0.05 (not significant), * *p* < 0.05, ** *p* < 0.01, *** *p* < 0.001.

To further investigate whether CA influences autophagic activity in YMECs, we evaluated autophagy-related responses. Immunofluorescence and Western blot assays further showed that LPS markedly elevated the LC3 II/I ratio compared to the control group, indicating enhanced autophagic activation. Pretreatment with 150 μM CA significantly reduced LC3 II accumulation (Figure 4E,F,J,K; *p* < 0.05). Furthermore, it was determined that LPS treatment led to a substantial upregulation of P62 and Beclin1 protein expression (Figure 4G–I; *p* < 0.05); however, CA pretreatment did not significantly alter the expression of these markers (Figure 4G–I; *p* > 0.05), implying a selective regulatory role in autophagy rather than a broad inhibition of autophagic pathways. As shown in Figure 5, *Nrf2* mRNA expression was significantly lower in the LPS-treated group compared with the controls, while 150 μM CA pretreatment strongly increased Nrf2 expression (*p* < 0.001). Furthermore, LPS significantly increased the expression of ER stress-related genes (*CHOP* and *GRP78*) and autophagy-related genes (*Atg5, Beclin-1*, and *LC3*), while CA pretreatment had the effect of attenuating these changes (*p* < 0.001).

We next examined the effects of CA on milk protein synthesis. As illustrated in Figure 5, LPS exposure significantly reduced β-casein and κ-casein levels compared to the control group (*p* < 0.05), while α-casein remained unaffected (*p* > 0.05). Pretreatment with 150 μM CA notably enhanced overall casein expression. Although LPS did not result in a substantial alteration in casein gene expression, CA pretreatment significantly upregulated the expression of the casein gene relative to the LPS-treated group (*p* > 0.05). Taken together, these results suggest that CA regulates autophagy-related pathways and contribute to maintaining epithelial secretory function under inflammatory stress.

### 3.5. Nrf2 Partially Mediates the Inhibitory Effect of CA on Excessive Autophagy

**Figure 6:**
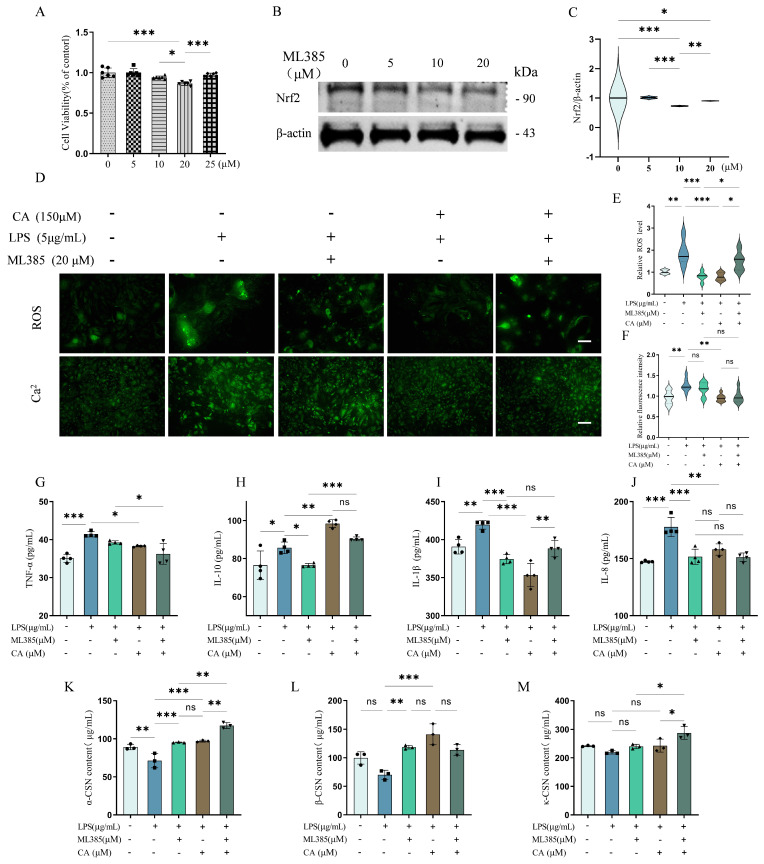
Inhibiting the Nrf2 signalling pathway modulates the inflammatory response to casein in LPS-induced primary YMECs. Cells were pretreated with 150 μM concentrations of CA for 6 h, followed by the addition of LPS (5 μg/mL) and ML385 for an additional 48 h. The total treatment duration was 54 h. (**A**) The effect of the specific Nrf2 inhibitor ML385 on the viability of YMECs. (**B**,**C**) Western blot analysis of Nrf2 protein. (**D**–**F**) ROS levels were quantified using DCFH-DA. Ca^2+^ staining images. Scale bar = 100 μm. Ca^2+^ concentration levels were quantified. (**G**–**J**) TNF-α, IL-1β, IL-10, IL-8 enzymatic activity content of immune factors (*n* = 3). (**K**–**M**) Quantification of α-CSN, β-CSN, and κ-CSN content (*n* = 3). One-way ANOVA, Dunnett’s post hoc test. Data are presented as the mean ± SEM. ns *p* > 0.05 (not significant), * *p* < 0.05, ** *p* < 0.01, *** *p* < 0.001.

To further elucidate the role of Nrf2 in CA-mediated protection, we employed ML385, a selective inhibitor of Nrf2 signaling. Treatment with 20 μM ML385 significantly reduced both cell viability and Nrf2 protein expression, whereas 10 μM had a milder effect (Figure 6A–C, *p* < 0.05). Therefore, 20 μM ML385 was used in subsequent experiments.

As expected, LPS exposure led to a pronounced increase in ROS levels and Ca^2+^ leakage (Figure 6D–F, *p* < 0.05). CA pretreatment effectively attenuated these effects. However, upon Nrf2 inhibition, ROS levels surged significantly, while Ca^2+^ leakage remained unchanged (Figure 6D–F, *p* > 0.05), indicating that oxidative stress is more sensitive to Nrf2 modulation than calcium dysregulation.

We next examined inflammatory cytokine production. Inflammatory cytokine analysis revealed that LPS significantly elevated TNF-α, IL-1β, IL-8, and IL-10 levels (Figure 6G–J, *p* < 0.05). CA pretreatment reduced the levels of pro-inflammatory cytokines (TNF-α, IL-1β, IL-8) and maintained IL-10 expression, consistent with its anti-inflammatory role. One should note that Nrf2 inhibition partially reversed these effects—most significantly, IL-1β secretion increased despite CA pretreatment (Figure 6H, *p* < 0.05).

**Figure 7:**
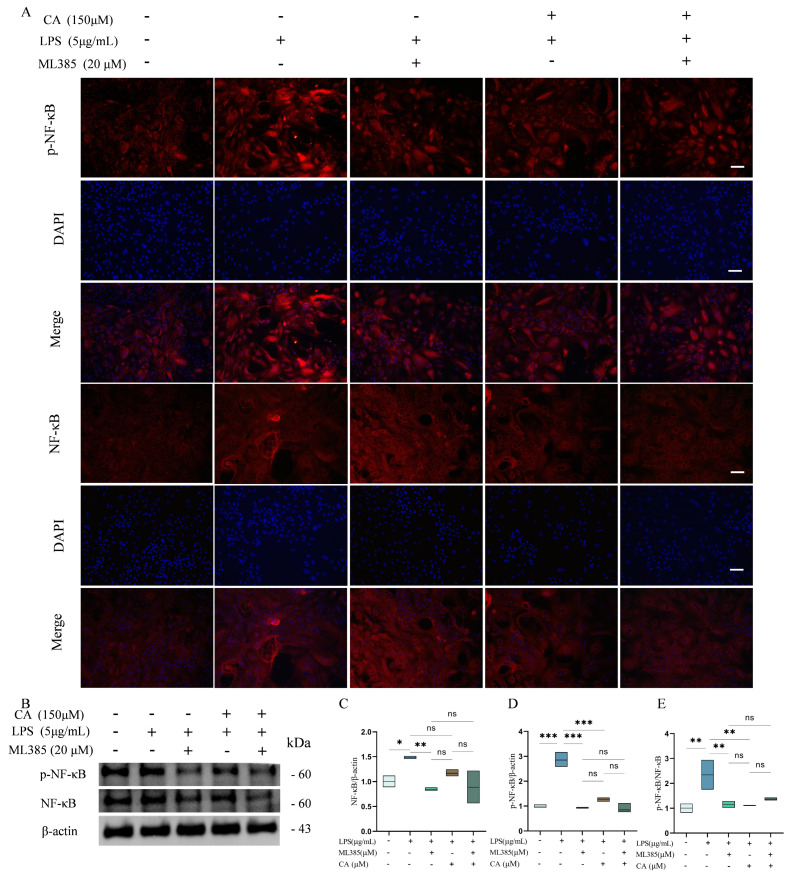
Inhibition of the Nrf2 signalling pathway modulates the effect of NF-κB on LPS-induced primary YMEC inflammatory responses in CA. Cells were pretreated with 150 μM concentrations of CA for 6 h, followed by the addition of LPS (5 μg/mL) and ML385 for an additional 48 h. The total treatment duration was 54 h. (**A**) Immunofluorescence result for p-NF-κB, NF-κB, Scale bar = 10 μm. (**B**–**E**) Western blot analysis of NF-κB, p-NF-κB protein. One-way ANOVA, Dunnett’s post hoc test. Data are presented as the mean ± SEM. ns *p* > 0.05 (not significant), * *p* < 0.05, ** *p* < 0.01, *** *p* < 0.001.

**Figure 8:**
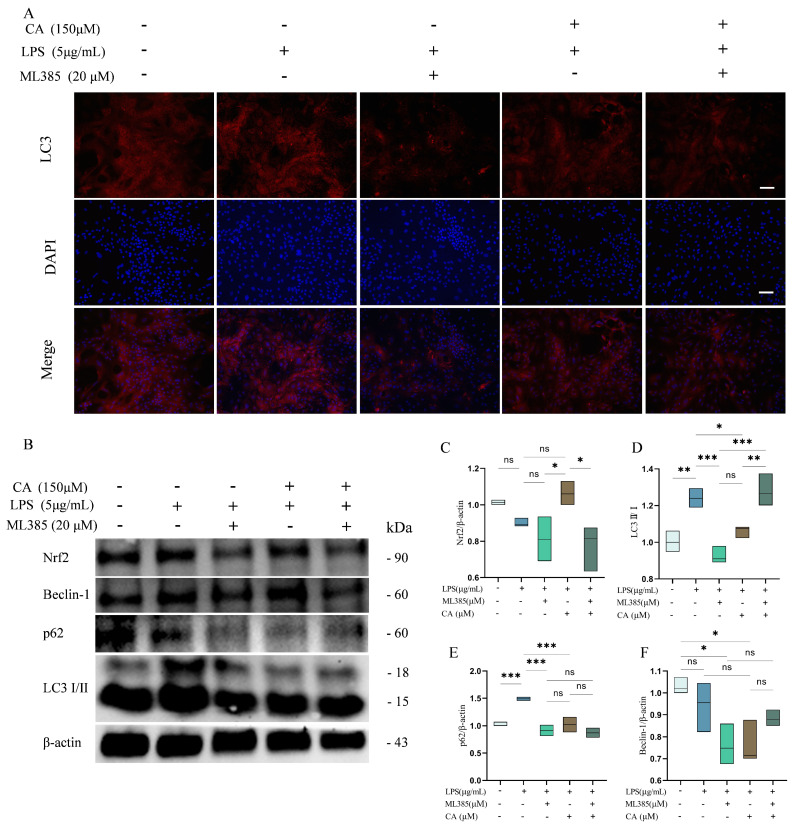
The effect of inhibiting the Nrf2 signalling pathway on alleviating the inflammatory response in primary YMECs induced by LPS. Cells were pretreated with 150 μM concentrations of CA for 6 h, followed by the addition of LPS (5 μg/mL) and ML385 for an additional 48 h. The total treatment duration was 54 h. (**A**) Immunofluorescence result for LC3, Scale bar = 10 μm. (**B**–**F**) Western blot analysis of Nrf2, Beclin-1, p62, and LC3 protein. One-way ANOVA, Dunnett’s post hoc test. Data are presented as the mean ± SEM. ns *p* > 0.05 (not significant), * *p* < 0.05, ** *p* < 0.01, *** *p* < 0.001.

In terms of casein synthesis, LPS reduced α-casein levels and trended toward lowering β-casein (Figure 6K–M, *p* < 0.05). CA pretreatment restored both α- and β-casein expression. Interestingly, co-treatment with ML385 and CA led to a further increase in α-casein and κ-casein levels, while β-casein remained similar to the CA-only group. Western blotting analysis showed that LPS-induced phosphorylation of NF-κB was significantly reduced by CA pretreatment or ML385 treatment alone (Figure 7A–E, *p* < 0.05). Importantly, co-treatment with ML385 and CA did not result in a further reduction in NF-κB activation compared with CA treatment alone. Finally, analysis of autophagy-related proteins revealed that LPS reduced Nrf2 expression and increased LC3-II and p62 levels (Figure 8A–F, *p* < 0.05). CA pretreatment restored Nrf2 levels and reduced LC3-II, p62, and Beclin-1 expression. However, when Nrf2 was inhibited by ML385, LC3-II levels rebounded significantly (Figure 8D, *p* < 0.05), while p62 and Beclin-1 remained unchanged. It is evident that the ML385 treatment did not lead to the abrogation of the inhibitory effect of CA on NF-κB activation.

## 4. Discussion

Mastitis remains a major challenge to yak milk production, with LPS from *E. coli* serving as one of the key pathogenic triggers [33]. Currently, the current therapeutic strategies for mastitis is focused on the administration of antibiotics, immunomodulatory therapies, and antioxidant therapies [34]. Nevertheless, the prolonged use of antibiotics has been demonstrated to induce issues related to antimicrobial resistance [35]. Consequently, the development of novel natural medicines with anti-inflammatory, antioxidant, and immunomodulatory functions has become a research focus for the treatment of mastitis. In particular, plant-derived polyphenols have shown considerable potential in mitigating inflammatory responses in mammary tissues [36,37,38,39,40]. That is why researchers are now turning to natural compounds. As a natural antioxidant and anti-inflammatory substance, the significant immunomodulatory and anti-inflammatory effects of CA have been confirmed in multiple studies [41,42,43]. In this study, we show that LPS triggers a multifaceted inflammatory cascade in YMECs. This is characterized by NF-κB activation, Nrf2 suppression, oxidative stress, ER stress, calcium dysregulation, excessive autophagy, and impaired casein synthesis. Our findings add further corroborate the anti-inflammatory effects of CA which disrupts this pathological axis by inhibiting NF-κB signalling pathways, restoring Nrf2 activity, mitigating reactive oxygen species accumulation, and modulating autophagy.

Our findings are consistent with previous studies in bovine and murine models, in which LPS-induced NF-κB activation was associated with increased pro-inflammatory cytokine production and ER stress [10,44,45]. The present study extends these observations to yak mammary epithelial cells, supporting the conserved nature of LPS-mediated inflammatory signaling across species. Consistent with its reported anti-inflammatory properties, caffeic acid (CA) suppressed NF-κB phosphorylation and reduced downstream cytokine production, in agreement with findings in endothelial and colonic epithelial cells [46,47]. However, unlike broad-spectrum anti-inflammatory agents, CA exhibited selective modulatory effects, attenuatingIL-1β and IL-8 at lower doses, while reducing TNF-α only at higher concentrations, suggesting context-dependent immunoregulatory effects.

An important finding of this study is that caffeic acid (CA) plays a regulatory role in autophagy. Previous studies have shown that LPS-induced cellular stress can alter the expression of autophagy-related proteins (such as LC3 and p62) through pathways associated with energy metabolism and inflammation [48,49]. Consistent with these findings, we observed that LPS-induced autophagy, as evidenced by an increased LC3-II/I ratio and elevated Beclin-1 and p62 expression, was significantly attenuated by CA pretreatment. Noteworthy, CA did not universally suppress autophagy; rather, it restored LC3-II levels to baseline without significantly altering Beclin-1 or p62 expression, indicating a nuanced modulation of autophagic flux rather than nonspecific inhibition. Further studies are needed to validate these observations using established approaches for monitoring autophagy, such as the use of lysosomal inhibitors [50]. This selectivity may reflect the ability of CA to restore autophagic balance under stress conditions, thereby preventing the detrimental consequences of excessive autophagy while preserving cytoprotective functions.

Nrf2 signaling contributes to the protective effects of caffeic acid under inflammatory stress. As a central regulator of antioxidant defense, Nrf2 is linked to autophagy through the p62–Keap1–dependent positive feedback loop, which connects oxidative stress and autophagy [51,52,53]. In the present study, CA increased Nrf2 expression and reduced intracellular ROS accumulation, accompanied by decreased LC3-II levels and lower production of inflammatory mediators. Pharmacological inhibition of Nrf2 using ML385 partially attenuated the antioxidant and autophagy-modulating effects of CA. Specifically, Nrf2 inhibition increased ROS accumulation and restored LC3-II levels despite CA pretreatment. However, inhibition of Nrf2 did not abolish the suppressive effect of CA on NF-κB activation. These findings suggest that Nrf2 functions as a modulatory factor that fine-tunes oxidative stress and autophagy, while NF-κB inhibition represents a parallel and predominant pathway. The persistence of CA-mediated anti-inflammatory activity under Nrf2 inhibition further supports the involvement of Nrf2-independent mechanisms, potentially including direct ROS scavenging or modulation of other redox-sensitive signaling pathways [11,48,54,55]. Moreover, considering the limited specificity and potential network effects of pharmacological Nrf2 inhibitors. To this end, future studies will employ Nrf2-targeted siRNA and evaluate canonical downstream markers, including HO-1 and NQO1, to more definitively determine the contribution of Nrf2 to CA-mediated regulation [52,53,54].

An unexpected finding of the present study was that Nrf2 inhibition increased α-casein and κ-casein expression, whereas β-casein expression remained unaffected. The paradoxical increase in casein upon Nrf2 inhibition suggests that compensatory signaling pathways were activated. The PI3K/Akt-mTOR/STAT5 axis, which is known to regulate milk protein synthesis [56,57]. excessive ROS accumulation is associated with suppressed casein synthesis in dairy cows [58], and our data suggest that Nrf2 inhibition may relieve certain transcriptional repressions under inflammatory stress, thus enhancing specific casein isoforms. Further investigation into phosphorylation states of STAT5 and mTOR, as well as promoter activities of casein genes, is warranted to clarify these compensatory mechanisms.

To summarize, this study provides the comprehensive evidence that caffeic acid mitigates LPS-induced inflammatory injury in yak mammary epithelial cells through a multi-targeted mechanism involving NF-κB inhibition, Nrf2 activation, and selective modulation of autophagy. These findings advance our understanding of yak mastitis pathogenesis and support the potential of CA as a natural agent for improving mammary health and milk quality.

## 5. Conclusions

**Figure 9:**
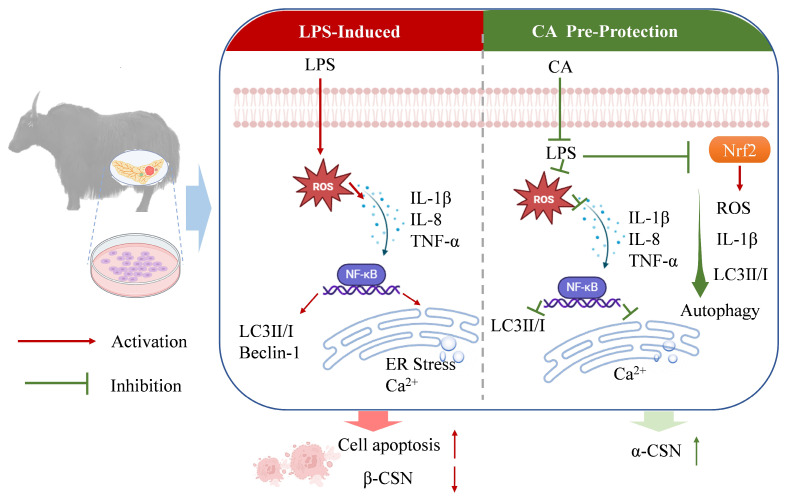
Working model of CA through NF-κB suppression regulates autophagy to counteract LPS-induced inflammatory damage in YMECs.

Together, our data provide new mechanistic insights into LPS-induced a coordinated inflammatory cascade in yak mammary epithelial cells, characterized by NF-κB activation, Nrf2 suppression, ER stress, Ca^2+^ leakage, excessive autophagy, and impaired casein synthesis. CA effectively counteracts this process by inhibiting NF-κB signaling, reactivating Nrf2, reducing ROS accumulation, and restoring autophagic homeostasis. Interestingly, Nrf2 inhibition only partially attenuated CA’s protective effects, suggesting that CA operates through both Nrf2-dependent and Nrf2-independent mechanisms (Figure 9). These findings identify CA as a mechanistically multi-targeted regulator capable of restoring epithelial homeostasis under LPS-induced inflammatory stress, and highlight its potential as a natural, sustainable intervention for mastitis prevention in yak dairy production systems.

## Figures and Tables

**Table 1:** Primer sequences and PCR conditions.

Gene	Primer Sequence ^1^ (5′ → 3′)	Annealing Temperature (°C)	Product Length (bp)
IL-1β	F: AACGTCCTCCGACGAGTTTC	60	89
	R: CCAGCACCAGGGATTTTTGC		
TNF-α	F: AGAGGGAAGAGCAGTCCCCA	58	125
	R: CGGAGAGTTGATGTCGGCTA		
IL-10	F: AGCACTACTCTGTTGCCTGG	58	98
	R: GGCTGGTTGGCAAGTGGATA		
IL-8	F: AACGAGGTCTGCCTAAACCC	60	199
	R: CCACACAGAACATGAGGCAC		
Nrf2	F: GCAGAGACATTCCCGTTTGT	60	99
	R: CCTGAGGAGGAGCAGTGAAG		
CHOP	F: TCTGGCTTGGCTTACTGAGG	60	153
	R: GACTGGCCACTCTGTTTCCG		
GRP78	F: CCTGTTCCGTTCCACCATGA	62	215
	R: CTTTCGTCAGGGGTCGTTCA		
Atg5	F: AGTTGCTCCTGAAGATGGGG	62	147
	R: TCTGTTGGTTGCGGGATGAT		
Beclin-1	F: GAAACCAGGAGAGACCCAGG	60	114
	R: GTGGACATCATCCTGGCTGG		
LC3	F: CCGACTTATCCGAGAGCAGC	60	161
	R: TGAGCTGTAAGCGCCTTCTT		
CSN1S1	F: TACCTGTCTTGTGGCTGTTGC	60	278
	R: CCTTTTGAATGTGCTTCTGCTC		
CSN2	F: AGTGAGGAACAGCAGCAAACAG	60	121
	R: AGCAGAGGCAGAGGAAGGTG		
CSN3	F: TTCAACTGCGGTCTAAATACTCTAAG	60	194
	R: TCAAAAAACTAAATCTGGCATAAAAG		
β-Actin	F: CACCAACTGGGACGACAT	60	202
	R: ATACAGGGACAGCACAGC		

^1^ (F) = forward; (R) = reverse.

## Data Availability

The original contributions presented in this study are included in the article. Further inquiries can be directed to the corresponding authors.

## Abbreviations

CA	Caffeic acid
YMECs	Yak mammary epithelial cells
CCK-8	cell counting kit 8
DAPI	4′,6-diamidino-2-phenylindole
DCFH-DA	dichloro-dihydro-fluorescein diacetate
DMEM	Dulbecco’s Modified Eagle Medium
FBS	fetal bovine serum
IL	interleukin
LC3	microtubule-associated protein light chain 3
NF-κB	regulating nuclear factor kappa-B
Nrf2	nuclear factor erythroid 2-related factor
TNFα	tumor necrosis factor alpha
ROS	reactive oxygen species
PVDF	polyvinylidene difluoride
ER-stress	endoplasmic reticulum stress
qRT-qPCR	quantitative real-time PCR
